# Supplementary material for: Nanofibrous Membrane with Encapsulated Glucose Oxidase for Self-Sustained Antimicrobial Applications
Source: Membranes (Basel). 2021 Dec 20;11(12):997. doi: 10.3390/membranes11120997 (PMC8704349; doi:10.3390/membranes11120997)
Supplement: Supplementary file 1 [file membranes-11-00997-s001.zip › Supplementary Materials.pdf]

membranes

Nanofibrous Membrane with Encapsulated Glucose Oxidase for Self-Sustained Antimicrobial Applications

Fernaldy Leonarta, Cheng-Kang Lee *

Department of Chemical Engineering, National Taiwan University of Science and Technology, Taipei 106, Taiwan, Republic of China
* Correspondence: cklee@mail.ntust.edu.tw

Supplementary Materials

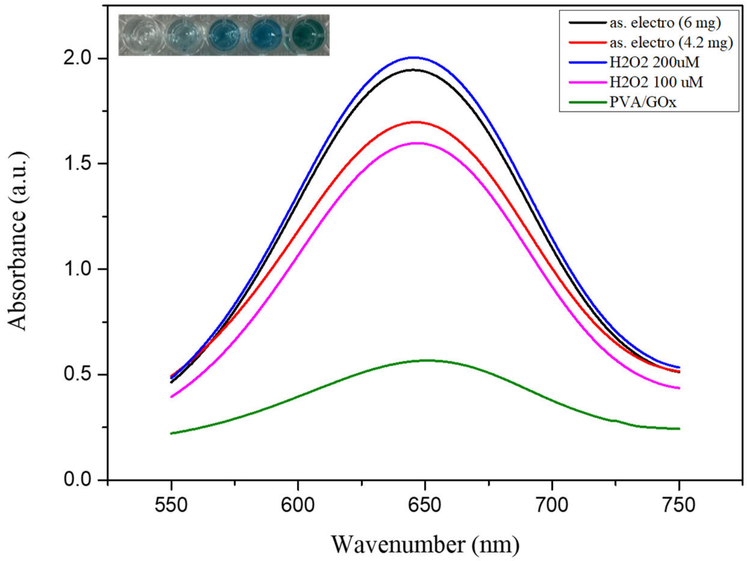

Figure S1 UV-vis absorption spectra of HRP-TMB solution containing different H_2O_2 concentrations and samples

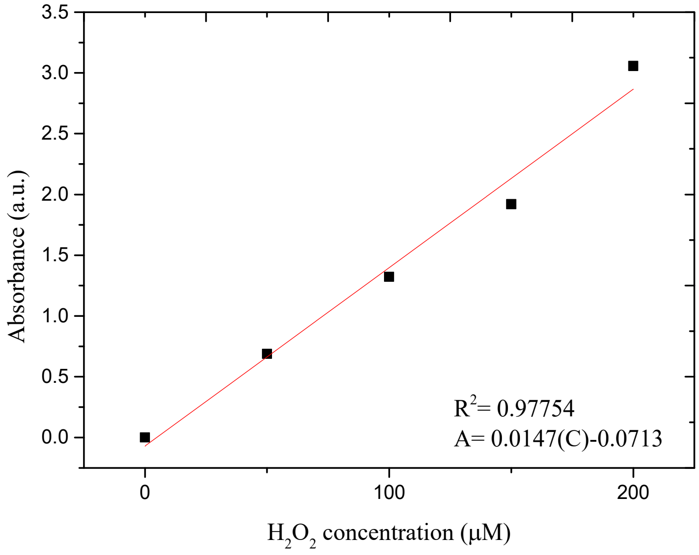

Figure S2 Standard curve showing the relation between H_2O_2 concentration and absorbance at λ=650nm

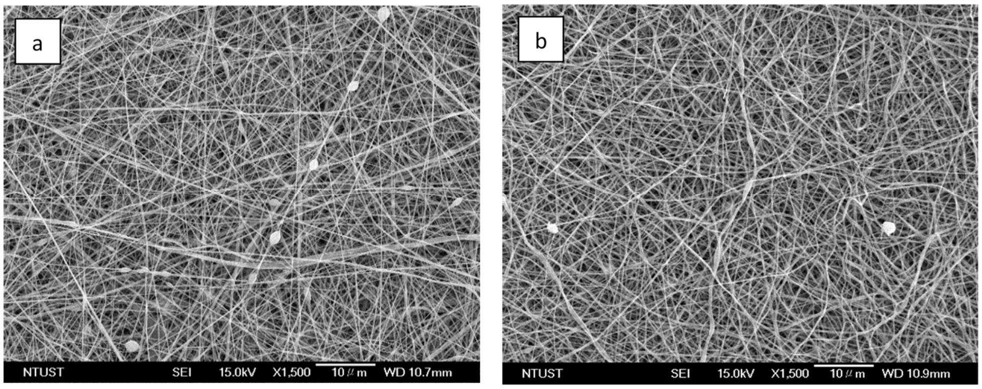

Figure S3 FE-SEM micrographs of simultaneously co-electrospun GOx/Glu nanofibrous membrane; (**a**): before GA vapor crosslinking, (**b**): after GA Vapor crosslinking under lower magnification
